# In vivo reflectance confocal microscopic findings in a case of trichofolliculoma^☆^

**DOI:** 10.1016/j.abd.2021.05.013

**Published:** 2022-01-14

**Authors:** Isil Karaarslan, Gokturk Oraloglu, Banu Yaman

**Affiliations:** a Department of Dermatology, Medical Faculty, Ege University, Izmir, Turkey; b Department of Pathology, Medical Faculty, Ege University, Izmir, Turkey

**Keywords:** Adnexal and skin appendage, Confocal microscopy, Dermoscopy, Neoplasms

## Abstract

Trichofolliculoma is a rare follicular hamartoma whose dermoscopic features have been scarcely reported. On the other hand, reflectance confocal microscopy features have not been described yet. In the present study, the authors report reflectance confocal microscopy features in a case of trichofolliculoma as squamous hyperplasia forming irregular finger-like protrusions around the hair follicle and papillomatous hyperplasia of the hair follicle epithelium, which correlated with histopathology. This case suggests that reflectance confocal microscopy may help incorrect in vivo diagnosis of trichofolliculoma in cases difficult to diagnose by morphology and dermoscopy.

## Case report

**Figure 1:**
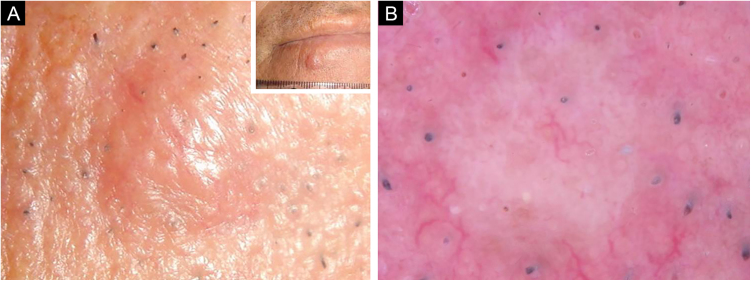
(A), A skin-colored, firm, non-tender papule with a diameter of 4 × 5 mm. (B), Whitish-pinkish homogenous structureless area on dermoscopy.

**Figure 2:**
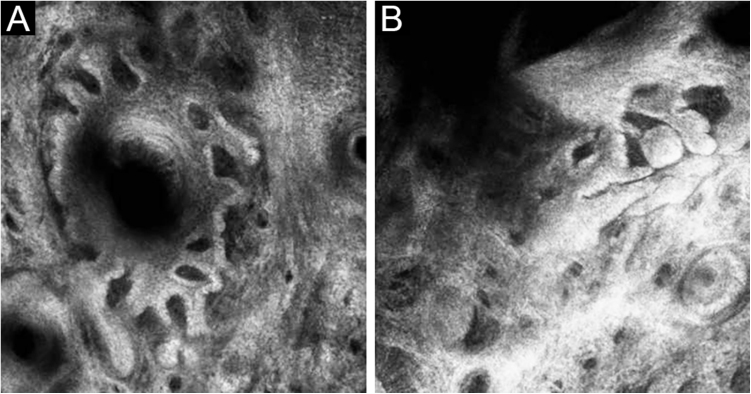
(A), Squamous hyperplasia forming irregular finger-like protrusions around the hair follicle (B), Papillomatous hyperplasia in the hair follicle epithelium on reflectance confocal microscopy.

**Figure 3:**
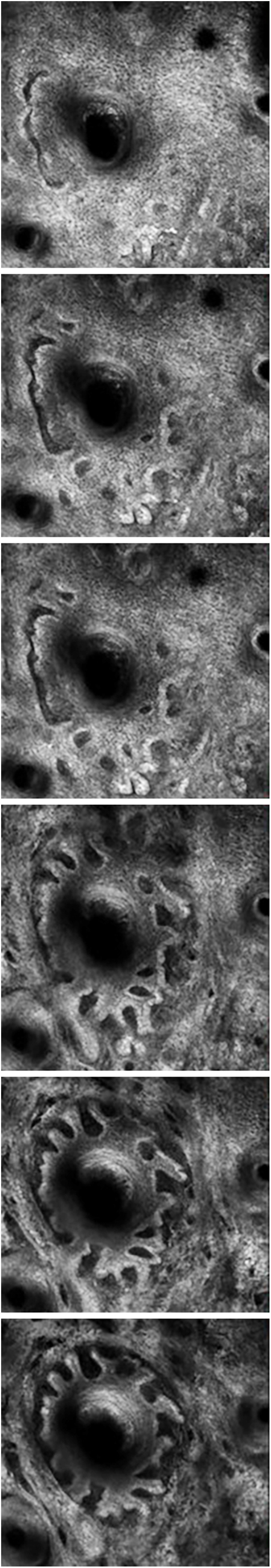
VivaStack imaging of the dilated follicule with finger-like protrusions around the follicle.

**Figure 4:**
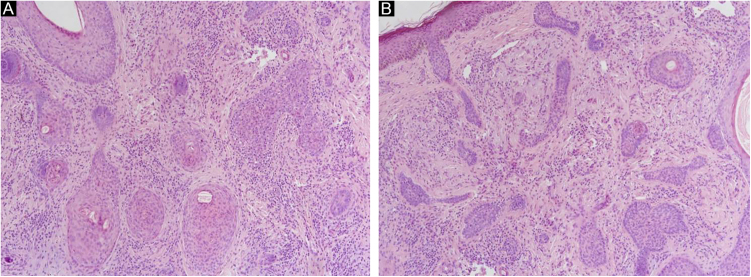
(A), Multiple radiate small abortive hair follicles of varying maturity degrees around central located primary follicle (Hematoxylin & eosin ×100). (B), Some abortive hair follicles located beneath the epidermis (Hematoxylin & eosin ×100).

A 66-year-old man was referred to our department because of an asymptomatic slowly growing papule on the chin with 1-year of duration. Physical examination revealed a skin-colored, firm, non-tender papule measuring 4 × 5 mm (Fig. 1A). The dermoscopic picture was non-specific with a whitish-pinkish homogenous structureless area. Prominent follicular openings and linear vessels on erythematous background were present on both lesional and peri-lesional sun-damaged skin (Fig. 1B). In Reflectance Confocal Microscopy (RCM) evaluation (Vivascope 3000 Handheld; Mavig GmbH, Munich, Germany), a honeycomb pattern with mild disarray was seen at the epidermal level. There were no atypical cells. There were some bright inflammatory cells. The epidermal thickness was attenuated, and at the Dermoepidermal Junction (DEJ) rings were few and far between. The authors observed squamous hyperplasia forming irregular finger-like protrusions around the hair follicle and papillomatous hyperplasia in the hair follicle epithelium was seen (Figure 2, Figure 3). At the dermal level, coarse collagen fibers beneath the follicles were observed. On histopathology, multiple radiate small abortive hair follicles of varying degrees of maturity around a few central dilated primary follicles filled with loose keratin were seen. The abortive hair follicles were secondary or tertiary follicles consistent with the diagnosis of a trichofolliculoma. The stroma between the follicles was cellular, vascularized with some lymphoid cells. Some of the abortive hair follicles located beneath the epidermis. The epidermis was thin and rete ridges were flattened (Fig. 4).

## Discussion

Trichofolliculoma is a rare follicular hamartoma mostly seen as a solitary asymptomatic, skin-colored, dome-shaped papule or nodule and usually affects the face and the scalp. The morphologic features of the lesion are generally non-distinctive unless it has a centrally located small tuft of hairs or a central pore.^1^ Histopathological appearance is characteristic of one or more primary central dilated follicle(s) and multiple secondary and tertiary hair follicles budding outward centrifugally from the central one.^2^

Correct clinical diagnosis of solitary non-pigmented lesions may be difficult, and in vivo diagnostic techniques have great importance in such cases. Dermoscopy has been widely used for decades in the the differential diagnosis of skin lesions, in most of which dermoscopic features have been described in detail. However, dermoscopic features of trichofolliculoma have been reported in only 3 cases until now.^3^, ^4^, ^5^ In these cases, central brown zone with several radial dark brown projections lying towards the periphery (“firework” pattern), fine peripheral serpiginous vascularization with centripetal disposition, and a white-pink central area with shiny white structures, dotted vessels and a central scale were the reported dermoscopic features. In the present case, a whitish pinkish homogenous structureless area was the only dermoscopic finding which was not helpful in the diagnosis.

RCM is another in vivo technique that has been relatively recently developed. It has advantages with giving cellular details of the upper layers of the skin and can be considered as “optical biopsy” of the living tissue. To the best of our knowledge, there has been no report on RCM features of trichofolliculoma.

In the present case, squamous hyperplasia forming irregular finger-like protrusions around the hair follicle and papillomatous hyperplasia in the hair follicle epithelium was the RCM findings that were concordant with histology. This case suggests that RCM may help incorrect in vivo diagnosis of trichofolliculoma in cases difficult to diagnose by morphology and dermoscopy.

## Financial support

None declared.

## Authors' contributions

Isil Karaarslan: Data collection, or analysis and interpretation of data; Manuscript or critical review of important intellectual content; Effective participation in the research guidance; Critical review of the literature; Final approval of the final version of the manuscript.

Gokturk Oraloglu: Data collection, or analysis and interpretation of data; Manuscript or critical review of important intellectual content; Critical review of the literature; Final approval of the final version of the manuscript.

Banu Yaman: Data collection, or analysis and interpretation of data; Manuscript or critical review of important intellectual content; Effective participation in the research guidance; Critical review of the literature; Final approval of the final version of the manuscript.

## Conflicts of interest

None declared.

## References

[1] McCalmont T.H., Pincus L.B., Bolognia J.L., Schaffer J.V., Cerroni L. (2018). Dermatology.

[2] Fulton E.H., Kaley J.R., Gardner J.M. (2019). Skin adnexal tumors in plain language: a practical approach for the general surgical pathologist. Arch Pathol Lab Med..

[3] Panasiti V., Roberti V., Lieto P., Visconti B., Calvieri S., Perrella E. (2013). The “firework” pattern in dermoscopy. Int J Dermatol..

[4] Jégou-Penouil M.H., Bourseau-Quetier C., Cajanus S., Rigon J.L., Risbourg M., Kluger N. (2015). Trichofolliculomes: revue rétrospective de 8 cas [Trichofolliculoma: a retrospective review of 8 cases]. Ann Dermatol Venereol..

[5] Garcia-Garcia S.C., Villarreal-Martinez A., Guerrero-Gonzalez G.A., Miranda-Maldonado I., Ocampo-Candiani J. (2017). Dermoscopy of trichofolliculoma: a rare hair follicle hamartoma. J Eur Acad Dermatol Venereol..

